# Charge structure in volcanic plumes: a comparison of plume properties predicted by an integral plume model to observations of volcanic lightning during the 2010 eruption of Eyjafjallajökull, Iceland

**DOI:** 10.1007/s00445-014-0828-4

**Published:** 2014-07-20

**Authors:** Mark J. Woodhouse, Sonja A. Behnke

**Affiliations:** 1 School of Mathematics, University of Bristol, University Walk, Bristol, BS8 1TW UK; 2 School of Geosciences, University of South Florida, 4202 E. Fowler Ave, Tampa, FL 33620 USA

**Keywords:** Volcanic lightning, Plume model, Charging mechanisms, Condensation

## Abstract

Observations of volcanic lightning made using a lightning mapping array during the 2010 eruption of Eyjafjallajökull allow the trajectory and growth of the volcanic plume to be determined. The lightning observations are compared with predictions of an integral model of volcanic plumes that includes descriptions of the interaction with wind and the effects of moisture. We show that the trajectory predicted by the integral model closely matches the observational data and the model well describes the growth of the plume downwind of the vent. Analysis of the lightning signals reveals information on the dominant charge structure within the volcanic plume. During the Eyjafjallajökull eruption both monopole and dipole charge structures were observed in the plume. By using the integral plume model, we propose the varying charge structure is connected to the availability of condensed water and low temperatures at high altitudes in the plume, suggesting ice formation may have contributed to the generation of a dipole charge structure via thunderstorm-style ice-based charging mechanisms, though overall this charging mechanism is believed to have had only a weak influence on the production of lightning.

## Introduction

Volcanic plumes transport large masses of ash and magmatic gases high into the atmosphere (Sparks et al. 1997). The dispersion of ash in the atmosphere over large distances can result in severe disruption to aviation (Miller and Casadevall 1999; Eurocontrol 2010), and sedimentation of ash can be hazardous to populations (Baxter 1999; Wilson et al. 2012). Real-time observations of the volcanic plume trajectory can assist in the response during volcanic crises and inform models used to forecast the ongoing hazard. Detection of volcanic lightning events (Hoblitt 1994; McNutt and Davis 2000; Thomas et al. 2007; Thomas et al. 2010; Bennett et al. 2010; Arason et al. 2011a; Behnke et al. 2013) could be used as a real-time remote sensing tool for determining the trajectory of volcanic plumes (Mather and Harrison 2006; James et al. 2008; Thomas et al. 2010).

Volcanic plumes are a multiphase mixture of solid particles, magmatic and atmospheric gases, and, in some conditions, liquid water and ice. Water vapour is the dominant component of the magmatic gas, with water vapour content of magmas in the range 3– 7 %wt in dacite, 1– 4 %wt in andesite and 1– 6 %wt in basalt (Wallace and Anderson 1999). Pyroclasts in volcanic plumes result from fragmentation of magma within the conduit (e.g., Wilson et al. 1980; Sparks et al. 1997; Woods 1998; Kaminski and Jaupart 1998) and erosion of the conduit wall (Wilson et al. 1980; Macedonio et al. 1994) and are transported within the plume until the vertical velocity of the gas falls below the particle settling velocity (Wilson and Walker 1987; Woods and Bursik 1991). Typically, the mass fraction of solids is high near to the vent, often in excess of 90 % (Woods 1988; Sparks et al. 1997), and erupted material is transported as a hot, dense momentum-driven jet (Sparks 1986). Entrainment, heating and expansion of atmospheric air can result in the mixture becoming buoyant before the momentum is exhausted and the erupted material then rises as a turbulent buoyant plume through the stratified atmosphere (Sparks 1986; Woods 1988; Sparks et al. 1997). Continued entrainment reduces the density contrast until the neutral buoyancy height is reached, where the density of the plume equals the atmospheric density. Inertia results in additional rise beyond the neutral buoyancy height, but the plume rapidly decelerates due to the reversal in the direction of the buoyancy force (Sparks 1986). At the plume top, where the vertical momentum vanishes, the plume is more dense than the atmosphere (Woods 1988; Sparks et al. 1997) and material slumps back and spreads laterally as a buoyancy-driven intrusion near the source (Bursik et al. 1992; Bursik 1998; Costa et al. 2013) and as an ash cloud further downwind(Bursik 1998).

In addition to the exsolved magmatic water in the plume, water vapour can be added at the source through magma–water interactions, at the expense of thermal energy (Wilson et al. 1978; Wohletz 1986; Mastin 1995; Koyaguchi and Woods 1996; Woods 1998; Van Eaton et al. 2012a), and by entrainment of atmospheric water vapour (Woods 1993; Glaze et al. 1997; Herzog et al. 1998). Large quantities of water vapour can be transported to high altitude by the relatively warm plume (Woods 1993; Sparks et al. 1997; Glaze et al. 1997; Herzog et al. 1998). Some of the vapour may condense as the plume cools to near-atmospheric temperatures, releasing latent heat and providing additional thermal energy to the plume (Woods 1993; Glaze et al. 1997; Herzog et al. 1998). Ice formation can occur if temperatures fall below the freezing temperature and sufficient ice nucleation sites are available (Herzog et al. 1998; Mastin 2007; Textor et al. 2006a, b; Durant et al. 2008; Van Eaton et al. 2012a). Ash particles are likely to provide plentiful ice nucleation sites (Durant et al. 2008). Ice alters the reflectivity of plumes and ash clouds (Guo et al. 2004) and therefore the water and ice content of volcanic plumes is an important consideration in forecasting or tracking (e.g. via satellite observations) volcanic ash.

The dispersion and deposition of ash is strongly influenced by the formation of aggregates (Carey and Sigurdsson 1982; Taddeucci et al. 2011; Bonadonna et al. 2011). Electrical charging of ash particles within volcanic plumes and clouds plays an important role in the process of aggregation (Gilbert et al. 1991; Gilbert and Lane 1994; Sparks et al. 1997), which impacts the lifetime of fine volcanic ash (< 63 microns diameter) in the atmosphere (Mastin et al. 2009; Brown et al. 2012) and associated sedimentation rates (James et al. 2008; Bonadonna et al. 2011; Taddeucci et al. 2011). The formation of dry aggregates in particular may rely on electrostatic forces to bind particles together (Sparks et al. 1997; James et al. 2002). Ice formation can alter the charge distribution, and also plays a direct role in wet aggregation processes (Durant et al. 2008; Durant et al. 2009; Van Eaton et al. 2012b). Knowledge of the charge distribution within volcanic plumes, together with the dynamics of the plume rise, is therefore necessary in order to accurately forecast atmospheric ash concentrations and the distribution of tephra fallout both near to the vent and at distallocations.

Lightning discharges are frequently observed during explosive volcanic eruptions, with lightning observed in eruptions columns that span a wide range of eruption magnitude (from VEI 1–6), plume height, volcano latitude, and magmatic composition (McNutt and Williams 2010). Lightning is most frequently observed in the convective part of the plume (Thomas et al. 2010; Behnke et al. 2014), although discharges from a drifting ash cloud more than 100 km from the vent were observed on two occasions at Redoubt (Hoblitt 1994; Behnke et al. 2009). While there is little direct hazard posed by volcanic lightning, the perceived hazard to local populations is significant and may alter the behaviour of communities during an eruption (Bird et al. 2011).

Several mechanisms can result in the charging of solid particles carried in volcanic plumes (Mather and Harrison 2006; James et al. 2008). Interactions between solid particles result in the development of a net charge carried by the particles through triboelectrical charging and fractoemission (Mather and Harrison 2006; James et al. 2008). The fracture charging mechanism may be a particularly important process for generating charge in explosive volcanic eruptions (James et al. 1998; 2000; James et al. 2008), where fragmentation processes in the conduit release charged particles and gases from the vent (Thomas et al. 2010). In experiments in which pumice samples are collided in a quiescent atmosphere, James et al. (2000) typically find a net negative charge is carried on pumice fragments with positively charged ions carried in the gaseous phase. However, a net positive charge is found on fragments from a sample of pumice with a low silica content (James et al. 2000), so the geochemistry of the erupted material may have an influence on the charge carried by the solid particles (James et al. 2008). From measurements of the atmospheric potential gradient at Sakurajima, Japan, Miura et al. (2002) infer a tripolar charge distribution which they attributed to size-segregated charging of particles and thus different rates of sedimentation for particles of different polarities. Size-segregated charge polarities have been observed in experiments where tribocharging of silicate beads produced a positive charge on large particles while small particles charge negatively (Forward et al. 2009; Lacks and Sankaran 2011). Furthermore, the particle size distribution has an important effect on the magnitude of the charge generated in triboelectric charging (Forward et al. 2009; Houghton et al. 2013; Cimarelli et al. 2014).

Thunderstorm-style charging mechanisms can also act in volcanic plumes (Mather and Harrison 2006; James et al. 2008; McNutt and Williams 2010). The formation of ice and graupel, and subsequent collisions, is thought to be responsible for the generation of charge in thunderstorm clouds (Takahashi 1978; Saunders et al. 2006). In this process, charge transfer results from collisions between light non-precipitating ice particles (ice crystals) and heavy precipitating ice particles (graupel) in the presence of supercooled liquid water (Pruppacher and Klett 1997; Saunders et al. 2006; Emersic and Saunders 2010). Laboratory experiments have shown that the charge polarity that is transferred to graupel depends on temperature and the amount of liquid water that is collected on the graupel particles through the process of riming (Saunders et al. 2006; Emersic and Saunders 2010). Ice formation in volcanic plumes can result in the development of charge at high altitude (Williams and McNutt 2005; Mather and Harrison 2006; James et al. 2008). The thunderstorm-style ice-contact charging mechanism (subsequently referred to as ice-based charging) has been suggested as an important component of the electrification of volcanic plumes during several eruptions (see e.g. Thomas et al. 2007, 2010; McNutt and Williams 2010; Bennett et al. 2010; Arason et al. 2011a; Behnke et al. 2013) but is not necessary in order to obtain volcanic lightning (Aizawa et al. 2010; Behnke et al. 2014; Cimarelli et al. 2014).

Since the transport of solid, gaseous and liquid phases within the turbulent buoyant eruption column is responsible for the separation of charge in the plume and the occurrence of lightning discharges, the relationship between plume dynamics and the occurrence of lightning suggests (1) lightning observations could be used as a test of the predictive ability of models to describe the trajectory of a volcanic plume in the atmosphere; (2) plume models could be used to infer the internal structure of volcanic plumes that cannot be measured directly, and so provide insights into the possible charging mechanisms occurring during eruptions. In this paper we address each of these, making use of observations from the second explosive phase of the 2010 eruption ofEyjafjallajökull.

This paper is organized as follows. We first discuss the lightning observations made during the 2010 eruption of Eyjafjallajökull (subsequently referred to as ‘the eruption’). An integral model of volcanic plumes is then introduced. We use the locations of very high frequency (VHF) sources detected by the lightning mapping array (LMA) in three dimensions to test the ability of the plume model to determine the trajectory and growth of the volcanic plume from Eyjafjallajökull. The predictions obtained from the plume model are used to examine the effects of changes in volcanic source and atmospheric conditions on the variation of lightning rates during the Eyjafjallajökull eruption. We then compare the model predictions of the moisture loading and temperature profile within the plume with the charge structure that can be determined from the LMA data. Finally we discuss the implications of our results and present some conclusions.

## Lightning observations during the 2010 eruption of Eyjafjallajökull

**Fig. 1:**
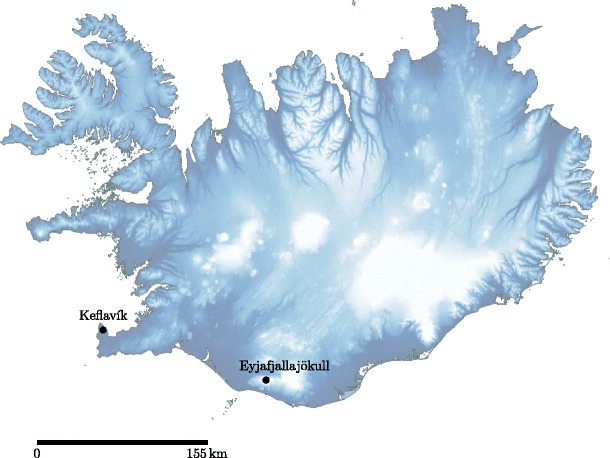
Location of Eyjafjallajökull and Keflavík in Iceland. The C-band weather radar at Keflavík is approximately 155 km from the volcano crater at the summit of Eyjafjallajökull

The summit eruption of Eyjafjallajökull, Iceland (Fig. 1), in April and May 2010 was a prolonged and sustained eruption of relatively small size (Gudmundsson et al. 2012). The eruption produced large quantities of fine ash, which were dispersed widely by atmospheric winds. Gudmundsson et al. (2012) identify four distinct phases of the eruption, including two explosive phases. The first explosive phase, 14–18 April, was the most vigorous and is characterized by phreatomagmatic activity as the erupted material melted glacial ice on the summit (Gudmundsson et al. 2012). The second explosive phase, 5–17 May, produced a similar amount of tephra as the first explosive phase (Gudmundsson et al. 2012), but over a significantly longer period of time. Mass eruption rates varied substantially during the second explosive phase (Gudmundsson et al. 2012), changing from periods of low activity where the source mass flux was an order of magnitude smaller than during the first explosive phase and periods of high activity where the source mass flux was comparable. The changes in source mass flux result in changes in the plume height (Arason et al. 2011b), although changing atmospheric conditions can also strongly influence plume height for weak volcanic eruptions and may dominate the variations in the recorded plume height at Eyjafjallajökull(Woodhouse et al. 2013).

Volcanic lightning was observed numerous times during the eruption. Two lightning detection systems were in use during the eruption: a long range, very low frequency lightning location network, ATDnet (operated by the UK Met Office), and a VHF lightning mapping array (LMA) for the detection of local lightning discharges.

**Fig. 2:**
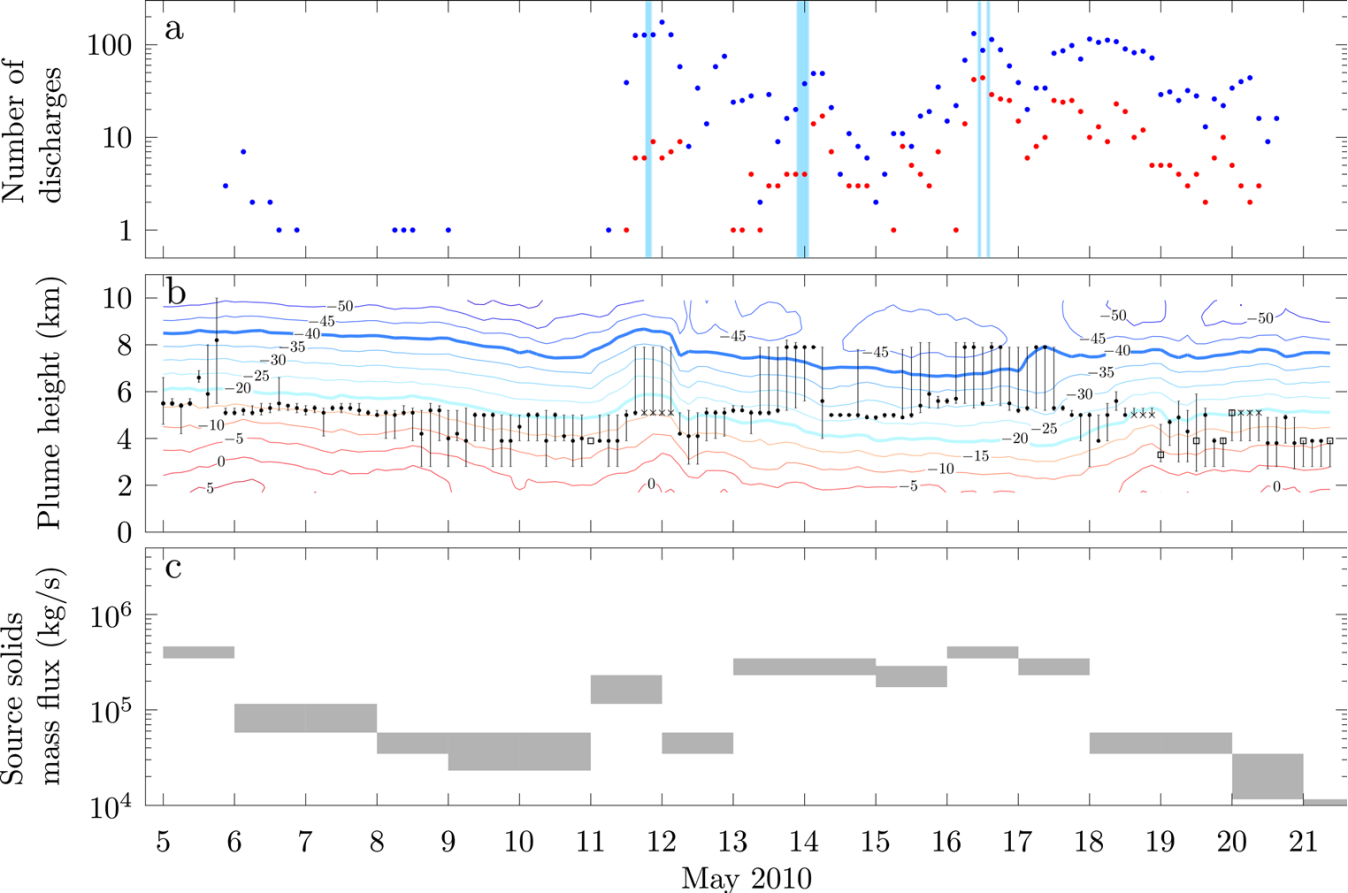
Time series of lightning events, radar-derived plume heights and estimates of the source mass flux of solids during the second explosive phase of the 2010 eruption of Eyjafjallajökull. (a) Number of lightning discharges in three hour intervals detected by the LMA (blue points) and ATDnet (red points). Blue shaded areas indicate time intervals where a negative-over-positive dipole charge structure can be inferred from the LMA data. (b) Plume height determined from radar observations. Black points denote median plume height in a three hour interval, with bars denoting the range of heights detected. Black squares denote intervals for which fewer than 10 height observations were obtained in the three hour interval, and black crosses denote occasions where radar observations are missing (here the heights recorded in the preceding interval are used in the plume model). Contours show isotherms of the atmospheric temperature, with the temperatures at which ice is expect to form and coexist with liquid water (−20°C) and at which ice is expected to occur without liquid water (−40°C) highlighted by thick lines. (c) The solids mass flux at the source estimated by Gudmundsson et al. (2012)

ATDnet was developed for meteorological applications and detects high current pulses from cloud-to-ground discharges and strong intracloud discharges (Bennett et al. 2010; Arason et al. 2011a). Therefore, only large volcanic lightning events are found while small but more frequent discharges are not detected (Behnke et al. 2014), although detection can occur over very large distances. Bennett et al. (2010) and Arason et al. (2011a) used ATDnet to detect lightning discharges in the volcanic plume from Eyjafjallajökull during the 2010 eruption, and found peak lightning discharge rates of 22 per hour on 16 May, 2010 (Fig. 2a).

The LMA is a multi-sensor array of receivers that can detect sources of impulsive VHF radiation, herein referred to as ‘VHF sources’, produced during electrical breakdown of air (Rison et al. 1999; Thomas et al. 2004). The LMA locates VHF sources from virtually all lightning discharges within its range, regardless of peak current, and thus can locate lightning discharges with smaller peak currents than ATDnet, such as frequent near-source volcanic lightning events (Behnke et al. 2014). Using time-of-arrival methods the VHF sources from lightning can be located in three-dimensions and sets of spatially and temporally correlated sources are combined to give a high resolution three-dimensional spatial and temporal lightning discharge map (Thomas et al. 2004). Here we consider discharges containing at least 10 correlated sources, which are referred to as regular discharges by Behnke et al. (2014). A temporary LMA consisting of six stations was installed in southern Iceland during the eruption (see Behnke et al. 2014, for full details) and was fully operational from 1 May 2010, providing three-dimensional spatial locations of lightning. Three-dimensional data was obtained during the last few days (1–5 May) of the effusive phase (18 April–5 May), throughout the second explosive phase (5–17 May) and during the declining phase of explosive activity (17–22 May). The LMA detected and located approximately 7700 discharges; the rate of regular discharges peaked at 67 per hour on 11 May 2010 (Behnke et al., 2014 and Fig. 2a).

### Lightning time series

During the second explosive phase of the eruption, two distinct periods of lightning activity were identified in both the ATDnet (Arason et al. 2011a) and LMA (Behnke et al. 2014) data sets. Between 5 and 10 May lightning observations were infrequent and sporadic, whereas from 11 to 21 May relatively high and sustained rates of lightning discharges were observed (Fig. 2a). In Fig. 2, the variation in the number of lightning discharges detected by the LMA and by ATDnet are compared to radar-derived plume height estimates, atmospheric temperature, and the source mass flux of solids as estimated by Gudmundsson et al. (2012) based on tephra deposits and plume height observations.

**Fig. 3:**
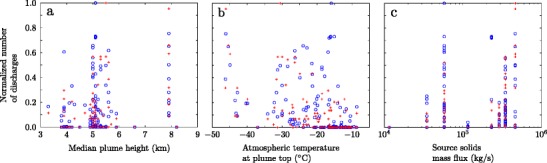
Number of lightning discharges detected by the LMA (blue circles) and ATDnet (red crosses) during the second explosive phase of the 2010 eruption of Eyjafjallajökull as a function of (a) the median plume height determined by the weather radar, (b) the atmospheric temperature at the median plume height, and (c) the upper bound of the Gudmundsson et al. (2012) estimates of the source solids mass flux. The number of lightning discharges has been normalized by the maximum number of discharges detected in a three-hour interval (44 for ATDnet, 175 for the LMA)

Arason et al. (2011a) show variations in the lightning discharge rates detected by ATDnet are correlated with the ambient atmospheric temperature at the plume top altitude (Fig. 3), with more frequent lightning occurring when ambient temperatures at the plume top are below −20°C. This is consistent with the expectation that substantial ice formation and mixed phase conditions occur when the temperature falls below −20°C and ice-contact charging mechanisms that operate in meteorological clouds become active (Takahashi 1978; Krehbiel 1986; Beard and Ochs 1986; Pruppacher and Klett 1997; Saunders et al. 2006) and thus leads to the hypothesis that ice-contact charging at high altitudes in the plume results in the lightning discharges (Arason et al. 2011a). Note, however, Arason et al. (2011a) do not assess the water content of the plume which is an essential constraint on the phase stability of water within the plume. The connection between lightning rates and atmospheric temperature cannot explain the sudden onset of lightning on 11 May, with the increase in plume height to altitudes above the −20°C isotherm occurring several hours after the onset of lightning (Fig. 2b), or the lightning occurring from 18 to 22 May when the plume height was most frequently below the −20°C isotherm (Fig. 2b), as recognized by Arason et al. (2011a). In addition, the plume height measurements are made using a weather radar and the uncertainty in the measurement often results in plume heights that span several isotherms(Fig. 2b).

In contrast to the ATDnet observations, LMA observations of flash morphology (Behnke et al. 2014) indicate that silicate-based charging was dominant and Behnke et al. (2014) saw no compelling evidence that atmospheric temperatures at or below ice-forming temperatures are connected to the occurrence of lightning (Behnke et al. 2014). Thus, if ice-contact charging was occurring, it was having a weak influence on the overall electrification of theplume.

The lightning rates observed by ATDnet show some correlation with plume height, with larger numbers of discharges occurring when the plume reaches higher altitudes (Fig. 3a and Arason et al. 2011a). As the source mass flux has a strong control on the plume height (Morton et al. 1956; Wilson et al. 1978; Sparks 1986; Woods 1988; Sparks et al. 1997; Mastin et al. 2009; Degruyter and Bonadonna 2012; Woodhouse et al. 2013) there is also a correlation between the number of discharges observed by ATDnet and the source mass flux estimated by Gudmundsson et al. (2012) (Fig. 3c). In contrast, the lightning rates observed by the LMA are uncorrelated with either the plume top height (Fig. 3a) or the source mass flux (Fig. 3c). The correlations between lightning rates and plume top temperature for lightning detected by ATDnet are not apparent in the LMA data (Fig. 3b), suggesting the large discharges detected by ATDnet are affected by ice-based charging, as suggested by Arason et al. (2011a), while the discharges resulting from vent and near-vent charging that are detected by the LMA are likely less influenced by atmospheric conditions (Behnke et al. 2014). However, as noted by Behnke et al. (2014), the LMA observations indicate that the discharges located by ATDnet initiated near the vent, and therefore the large discharges occur as a result of high charge concentrated near the vent rather than ice-based charging at high altitude in the plume.

### Plume charge structure analysis

The signals obtained by the LMA can be analyzed to infer the charge structure within the plume using methods that are well-established in studies of thunderstorms (Thomas et al. 2002; Marshall et al. 2005; Rust et al. 2005; Wiens et al. 2005; Tessendorf et al. 2007; Krehbiel et al. 2008; Bruning et al. 2010). Behnke et al. (2014) applied the charge structure analysis to the LMA data from the 2010 eruption of Eyjafjallajökull and find the charge structure is predominately a positive-charge monopole (i.e. lightning was propagating into regions of net positive charge) during the second explosive phase of the eruption. However, on a few occasions (as indicated in Fig. 2a) a negative-over-positive charge dipole structure can be inferred (Behnke et al. 2014). As the charge structures are determined by analysis of the temporal development of discharges, additional regions of charge may exist for which electrical breakdown did not occur (Behnke et al. 2014). Therefore the inferred charge structure does not preclude the existence of additional, electrically inactive charged regions. Below we compare model predictions of the temperature and water content in the plume with the charge structure determined from the LMA observations to examine the role of ice formation in the generation of negatively charged regions at high altitude in the plume.

## Methods

### Integral models of volcanic plumes incorporating meteorological data and phase change of water

Detailed modelling of volcanic plumes is challenging due to the turbulent and multiphase character of eruption columns (Sparks et al. 1997). Numerical models that attempt to resolve the turbulent structure (Valentine and Wohletz 1989; Dobran et al. 1993; Oberhuber et al. 1998; Suzuki et al. 2005; Ogden et al. 2008) and microphysical processes occurring within volcanic plumes (Herzog et al. 1998; Textor et al. 2006a, b) have been developed, but the computational resources required currently precludes the use of these models for rapidly simulating plume dynamics during volcanic crises.

Integral models of turbulent buoyant plumes (Morton et al. 1956) that describe steady plume dynamics have been used to model plumes in industrial and environmental settings (Woods 2010). The turbulent entrainment is modelled using simple parameterizations that relate the entrainment velocity to the bulk velocity of the plume (see e.g. Morton et al. 1956; Hewett et al. 1971; Turner 1986; Carazzo et al. 2006). The multiphase character of volcanic plumes can be incorporated into integral models (Woods 1988; Glaze and Baloga 1996; Sparks et al. 1997), and phase changes of water (Woods 1993; Glaze et al. 1997; Mastin 2007) and the effect of wind (Bursik 2001; Bursik et al. 2009; Degruyter and Bonadonna 2012; Woodhouse et al. 2013; Devenish 2013) can be included.

Recently, Woodhouse et al. (2013) developed an integral model of volcanic plumes that incorporates detailed meteorological profiles, including wind and moisture content, using the entrainment formulation of Hewett et al. (1971) (see also Degruyter and Bonadonna 2012, Devenish 2013, Mastin 2014). We adopt this model here. The governing equations used in this study are given in Appendix and full details of the integral model are given in Woodhouse et al. (2013).

The integral model (Woodhouse et al. 2013) includes a description of the transport of water vapour and condensation to liquid, and the release of latent heat on condensation. Given the abundance of fine ash particles in the volcanic plume, it is thought that cloud condensation nuclei are readily available (Woods 1993; Williams and McNutt 2005; Durant et al. 2008) and the model assumes condensation occurs immediately once the gaseous phase becomes saturated with respect to water vapour (Woods 1993). However, the formation of ice is not explicitly included in the model. The thermodynamics of ice formation in clouds is complicated, as supercooled water and ice can coexist over a range of temperatures (Rogers and Yau 1989; Pruppacher and Klett 1997). The proportion of ice particles in a mixture of water vapour, liquid water and ice is not only temperature dependent, but is determined by the availabilityof ice nucleation sites and the growth of ice crystals by diffusion and accretion to form graupel (Rogers and Yau 1989; Pruppacher and Klett 1997). An additional complication is the dependence of the freezing temperatures on the physical properties and, to a lesser extent, the chemical composition of the ash acting as ice nucleation sites and the presence of volatile chemical species, as described by Raoult’s Law (Pruppacher and Klett 1997). As the latent heat released in freezing of liquid water is an order of magnitude smaller than the latent heat of condensation, the neglect of freezing of water to ice will have only a small effect on the energy balance in the plume (Woods 1993; Herzog et al. 1998). For very large eruptions, where water vapour is transported to stratospheric altitudes (Woods 1993; Glaze et al. 1997), we may additionally require a description of deposition freezing (the phase change of water vapour directly to ice). However, for the relatively low plumes observed during the 2010 eruption of Eyjafjallajökull, we expect the condensation of water vapour to liquid water and subsequent freezing to dominate, and the latent heat released during condensation to be the leading order thermodynamic effect of the phase change.

Without a detailed model of ice formation we can nevertheless anticipate the formation of ice in the plume from the condensation of water vapour to liquid water; if the model predicts the existence of liquid water in a region colder than −20°C we expect that a significant fraction of this water will freeze to ice (Durant et al. 2008). If the temperature in the plume is below −40°C we expect no liquid water as all condensed water droplets will freeze spontaneously (Rogers and Yau 1989; Pruppacher and Klett 1997).

As an example of the small effect of ice formation on the energy budget of the plume we consider the plume from Eyjafjallajökull at 2100 (note all times given are UTC) on 13 May 2010, when our model predicts the highest mass fraction of condensed water over the course of the second phase of the eruption to occur. The weather radar gives a maximum plume height of 8.1 km and the atmospheric temperature falls below −20°C, so substantial ice formation is expected. Our model predicts that condensation contributes a maximum of ∼1.3% to the total energy flux of the plume from the release of latent heat of condensation. If instead we assume that, once saturated, the water vapour instantaneously changes phase through direct deposition to form ice, then the release of latent heat of deposition would provide a maximum of ∼1.7% to the total energy flux of the plume. This is an upper bound on the contribution of ice formation to the energy budget of the plume since we expect freezing of liquid water rather than deposition freezing to be the dominant ice forming process unless the temperature falls below −40°C (Rogers and Yau 1989; Pruppacher and Klett 1997). These estimates of the small thermal energy contribution of ice formation are consistent with results from numerical experiments obtained by Herzog et al. (1998) using a model that describes microphysical processes, and suggest that neglecting ice formation does not significantly alter the modelled thermodynamics (see also Woods 1993). We note that ice formation will have a dynamical effect through the lower density of ice in comparison to liquid water. However, since the condensed water or ice content in volcanic plumes is small (typically less than 1.5 g/kg for the eruption conditions we consider), we expect the differences in the density of condensed water phases to have a negligible effect on the bulk density of theplume.

### Modelling assumptions and limitations

**Fig. 4:**
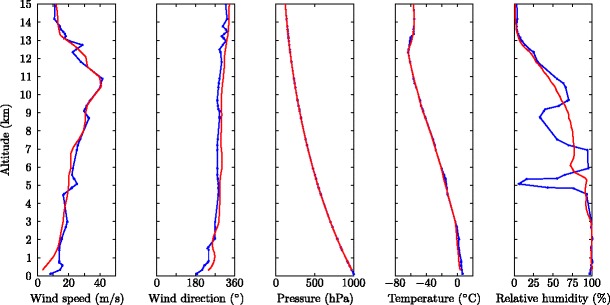
Meteorological profiles at 0000 on 12 May 2010 from radiosonde measurement at Keflavík (blue line) and from the Met Office Unified Model interpolated to Eyjafjallajökull (*red line*)

In order to make predictions using the integral plume model, meteorological conditions and volcanological source conditions are required as inputs, both of which are subject to observational uncertainty. As detailed direct meteorological observations at Eyjafjallajökull are not available for the 2010 eruption we use meteorological data from the Met Office Unified Model (data provided by the U.K. Met Office from the Unified Model global data archive). The meteorological profiles at Eyjafjallajökull during the eruption are approximated by interpolating spatially and temporally the Unified Model data using the Met Office NAME atmospheric dispersion model. While the use of numerical weather prediction (NWP) model data rather than direct observations of atmospheric profiles could lead to discrepancies between model predictions and observations, the profiles obtained by interpolation of the NWP data compare well with radiosonde soundings taken at Keflavík twice daily (e.g. Fig. 4 and Arason et al. 2011a; Woodhouse et al. 2013).

Our model adopts meteorological profiles at a single location, taken to be the summit of Eyjafjallajökull (63.63 N, 19.62 W). Therefore, while vertical variations in atmospheric conditions are described, our model does not account for changing atmospheric conditions with lateral distance from the vent. As we are primarily concerned with near-source processes, within a lateral distance of approximately 30 km from the vent, we expect only slight lateral variation in the atmospheric conditions at high altitude, although topographic effects could affect the meteorological profiles at lower levels. Furthermore, as Eyjafjallajökull is located near to the Icelandic coast, northerly winds blow the plume over the sea where the wind and moisture loading of the atmosphere will differ from profiles on land. The single location meteorology is sufficient for our study of near-source plume dynamics. Indeed, the integral model is not expected to be an appropriate description of the plume dynamics far downstream where the motion is predominately horizontal.

Throughout this study, source conditions at the volcanic vent are estimated by matching the model predicted plume height to observations of the plume height made by a C-band weather radar at Keflavík International Airport, 155 km west of Eyjafjallajökull (Arason et al. 2011b). The model predictions are therefore determined independently from the lightning observations. The distance from Keflavík to Eyjafjallajökull, together with the scanning strategy employed during the eruption, lead to semi-discrete jumps in the radar plume heights (Arason et al. 2011b). Furthermore, the minimum detectable reflectivity of the C-band radar may exceed the reflectivity response of low concentrations of fine ash at a distance of 155 km from the radar detector (Marzano et al. 2006; Marzano et al. 2011), and the formation of hydrometeors in the plume will significantly alter the reflectivity (Rogers and Yau 1989; Guo et al. 2004; Durant et al. 2009). Therefore, there are significant uncertainties in the plume height estimates, as indicated by bars showing the range of the inferred plume height in three-hour intervals on Fig. 2b. In addition, some time intervals do not have associated radar observations due to the plume being obscured by precipitating clouds, missing data, or changes in the operational mode of the radar (Arason et al. 2011b). When radar observations are missing from the dataset, we take the pragmatic approach of using the nearest preceding radar observation motivated by operational uses of radar observations. While other interpolants could be used, for example time-weighted averages of neighbouring observations, the large and abrupt changes in the radar plume heights (Fig. 2b) suggest no interpolant is to be preferred.

To account for the uncertainty in the plume heights, model calculations adopt the median plume heights determined over 3-hour intervals and, additionally, the maximum and minimum heights detected during the interval. The source velocity of erupted material is varied in the range 10– 200 m/s in order to match the model prediction of the maximum height of the plume centreline to the radar-derived plume height, while other source conditions are held fixed. Since the source temperature and volatile content of the erupted material is not known, we employ three source parameter sets, given in Table 1, representing an eruption with little addition of external water (subsequently referred to as ‘hot and dry’ conditions), an eruption with substantial external water added at the source and therefore a lower source temperature (subsequently referred to as ‘cool and wet’ conditions), and an intermediate case (subsequently referred to as ‘intermediate’ conditions). The radius of the plume at the source is taken to be 30 m throughout, based on observations of the Eyjafjallajökull crater following the 2010 eruption (Ripepe et al. 2013). While the appropriate length scale for the plume at the vent may differ from the crater size, the results are not greatly affected by the choice of radius of the plume at the source as, for buoyant plumes, increasing the source radius can be compensated by decreasing the source velocity since the source mass flux has the dominant control on the plume height (Sparks et al. 1997).

**Table 1:** Eruption source condition scenarios

Scenario	Source temperature *T*_0_	Magmatic water content *n*_0_
Hot and dry	1100 K	0.02
Intermediate	1000 K	0.05
Cool and wet	800 K	0.10

We note that it is not always possible to match modelled plume heights to the radar determined plume heights for all of the source conditions (Table 2). This is due to either the plume height observed by the radar being below or above the height of buoyant plumes predicted by the model for a plausible range of source velocities. For example, when hot and dry conditions are used, the model predicts that buoyant plumes frequently rise higher than the minimum height in the radar record (Table 2). In contrast, when cool and wet source conditions are used, the maximum height in the radar record is often in excess of the predicted maximum plume height (Table 2).

**Table 2:** Failure rate for matching modelled plume heights to the minimum, median and maximum of the radar determined plume heights for 3-hour intervals during 5–21 May 2010

	Radar derived plume height		
Source scenario	Minimum	Median	Maximum
Hot and dry	70 %	49 %	31 %
Intermediate	30 %	12 %	15 %
Cool and wet	26 %	31 %	54 %

## Results

### Plume trajectories

**Fig. 5:**
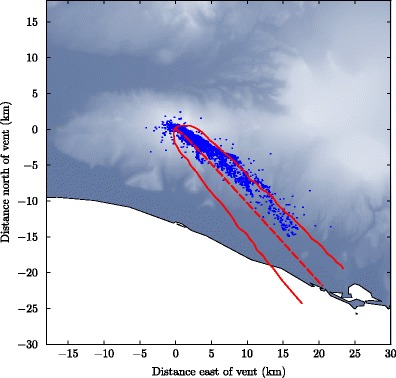
Plan view of the predicted centreline trajectory (red dashed line) and width (red solid line) of the plume from Eyjafjallajökull on 11 May 2010 at 2100 UTC, and located VHF sources from lightning detected by the LMA for the period 1900–2100 (blue points). Each VHF source detected by the LMA represents a piece of a lightning discharge event. Some of the scatter in the lightning observations is due to measurement noise. The vent is located at approximately 63° 37^′^ 29^″^ N, 19° 37^′^ 52^″^ W

**Fig. 6:**
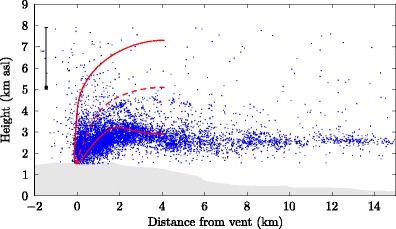
Side view of the predicted centreline trajectory (red dashed line) and upper and lower plume edges (red solid lines), up to the maximum rise height, of the plume from Eyjafjallajökull on 11 May 2010 at 2100 UTC and located VHF sources from lightning detected by the LMA for the period 1900–2100 (blue points). Each VHF source represents a piece of a lightning discharge. Some of the scatter in the VHF sources is due to measurement noise. As no radar observations are available during this period, we use the median (black circle, 5.1 km) and range (black line, 5.0 – 7.9 km) of radar determined plume heights for the closest preceding period, 1200–1500 on 11 May

An example of the predicted trajectory of the plume from Eyjafjallajökull on 11 May 2010 at 2100, determined from the plume model using the ‘intermediate’ source conditions, is shown in Figs. 5 and 6 together with LMA lightning observations for the period 1900 – 2100. In Fig. 5 the model calculation is extended to a distance of approximately 30 km from the vent, whereas in Fig. 6 the plume model calculation is terminated at the maximum rise height. The VHF sources show a clear trajectory towards the southeast, with lightning extending beyond 20 km from the vent (Fig. 5), suggesting the plume trajectory was strongly affected by the northwesterly winds. The VHF sources span a range of altitudes from the vent (at approximately 1.5 km) to 7.9 km (Fig. 6). During this period the median plume-top height determined from the weather radar was 5.1 km, and the maximum height was 7.9 km (Arason et al. 2011b). Qualitatively similar results are obtained using the alternative eruption source scenarios (Table 1).

### Variation in plume properties during the eruption

**Fig. 7:**
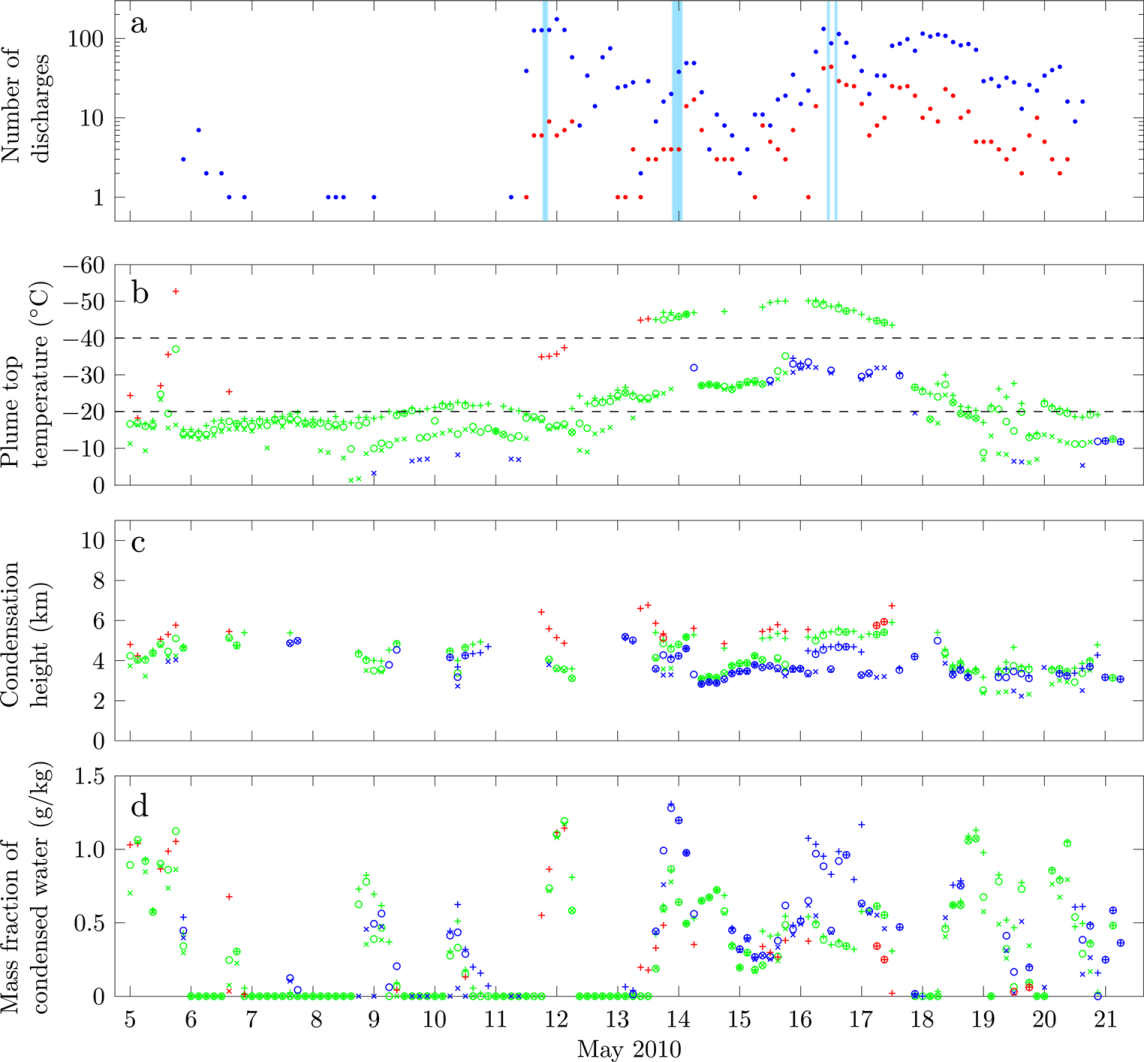
Time series of lightning events and model predictions of plume properties during the second explosive phase of the 2010 eruption of Eyjafjallajökull. (a) Number of lightning discharges in three hour intervals detected by the LMA (blue points) and ATDnet (red points). Blue shaded areas indicate time intervals where a negative-over-positive dipole charge structure can be inferred from the LMA data. (b) Temperature at the plume top predicted by plume model. Note the inverted temperature scale. The temperatures at which ice is expect to form and coexist with liquid water (−20°C) and at which ice is expected to occur without liquid water (−40°C) are marked. (c) The height at which condensation occurs in the plume as predicted by the integral plume model. Where data points are absent, no condensation is predicted to occur. (d) The maximum mass fraction of liquid water in the plume as predicted by the plume model. In (b)–(d) model predictions with source conditions determined using the median plume height, maximum plume height (+ ) and minimum plume height (×) are shown. Green points denote predictions using ‘intermediate’ source conditions. Model predictions using the alternative source conditions are shown, with red points denoting the ‘hot and dry’ source conditions and blue points denoting ‘cool and wet’ source conditions, where the predictions differ significantly from the predictions with ‘intermediate’ conditions

Here we reanalyze the variation in lightning rates using an integral plume model to assess the effect of changing conditions within the plume, in addition to atmospheric conditions. Figure 7 shows the variation in lightning rates together with the model predictions of the plume top temperature, the height at which condensation first occurs and the maximum mass fraction of condensed water in the plume over the course of the second explosive phase of the eruption.

In Fig. 7b–d model predictions using the ‘intermediate’ source conditions are shown. Predictions using the alternative ‘hot and dry’ or ‘cool and wet’ source conditions are often similar to the predictions using the ‘intermediate’ conditions and we therefore only plot these predictions where there is a significant difference. In particular, results using ‘hot and dry’ or ’cool and wet’ conditions are shown only when the plume top temperature differs by more than 10% (Fig. 7b), the condensation height differs by more than 200 m (Fig. 7b), and the maximum condensed water content differs by more than 20% (Fig. 7c). Note these values are arbitrarily selected.

The plume top temperature is insensitive to the source conditions, but is strongly dependent on the height of the plume since the temperature at the plume top is controlled by the atmospheric temperature. The condensation height is sensitive to both the plume height and the source conditions, with condensation occurring at higher altitudes when the plume ascends higher into the atmosphere and when ‘hot and dry’ conditions are used. In contrast, the condensation height is lower when the ‘cool and wet’ conditions are used since the water vapour content of the plume at the source is increased. The amount of condensed water in the plume is sensitive to the source conditions, with increased condensation when external water is added at the source, and to the plume height, with increased condensation when the maximum radar-derived plume height is used. However, the occurrence of condensation (i.e. the transition from an unsaturated plume to a plume where some condensation occurs) is relatively insensitive to the source conditions or plume height.

### Plume properties and charge structure

We examine the inferred charge structure together with vertical profiles of plume properties as predicted using the plume model, focusing on three occasions where a negative-over-positive dipole structure is observed (11 May, 1900–2100; 13 May, 2200–0000; 16 May, 1400–1600; see Behnke et al. 2014 for further details of these intervals), and three occasions where a positive monopole charge structure is inferred (12 May, 0300–0500; 12 May, 2100–2300; 17 May, 1200–1400). In all cases there are many VHF sources that are not assigned a charge polarity in the charge analysis. The charge analysis method is manual and is only applied to the larger discharges that show obvious channel structure. Even though many VHF sources remain undetermined, Behnke et al. (2014) found no compelling evidence of an electrically active upper negative region in the positive monopolecases.

In Fig. 9 we show comparisons of the charge structure inferred from the LMA with the model profiles of condensed water content and plume temperature on occasions when a dipole structure was observed. Model calculations at the mid-point of the interval over which charge analysis was performed are presented; these profiles are representative of the profiles calculated at other times during the interval. The charge analysis in Fig. 9 shows a clear separation of the charge regions, with negative charge regions localized at high altitude in the plume. On each occasion the model predicts substantial condensation of water vapour. In contrast, Fig. 10 shows comparisons of the inferred charge structure to plume model predictions on occasions when a monopole structure was observed. The temperature profiles in Figs. 9 and 10 show undercooling of the plume near the plume top height.

## Discussion

### Comparison of trajectories observed by the LMA with prediction from the integral model

The model trajectory in plan view (Fig. 5) matches the LMA-derived discharges quite well (Fig. 5), particularly near the vent. The model trajectory has some curvature near the vent during the initial predominantly vertical rise of the plume as the wind direction varies with altitude. This curvature can also be seen in the LMA observations (Fig. 5). However, as the plume model approaches the neutral buoyancy height and the motion becomes predominately horizontal, and interpolated meteorological profiles at a single location (above the vent) are used, there is little variation in the direction of the plume centreline. Therefore, far downwind of the vent, the model is unable to capture the additional curvature of the plume derived from the LMA observations.

The prediction of the plume width obtained from the integral model (Fig. 5) gives a reasonably good envelope ofthe VHF sources. However, the model cannot describe the VHF sources upwind of the vent, and the deviation from the observations is pronounced far downwind. The discrepancies may be due to limitations of the meteorological data, inparticular a difference in the wind direction obtained from the NWP with respect to the actual wind field leading to the apparent off-set of the modelled plume trajectory from the VHF sources, in addition to simplifications in the derivation of the model.

The side view of the plume model prediction (Fig. 6) shows a high density of VHF sources occurring on the lower plume edge as the plume bends over and begins to move predominately horizontally away from the vent. This is consistent with the expectation that pyroclasts ejected from the volcano carry charge and therefore localized charge separation occurs on the lower plume boundary as the larger particles begin to fall out of the plume. Ice-based charging is unlikely to play a role in the localization of VHF sources on the lower plume boundary as the temperature here remains above the freezing temperature.

The plume model uses wind data at the altitude of the plume centreline, which can be more than a kilometer above the lower edge of the plume downwind of the vent, while the VHF sources detected by the LMA are clustered on the lower plume edge. Therefore, varying wind direction with height is likely to result in a discrepancy between the model trajectory and the trajectory derived from LMA observation as pyroclasts falling out of the plume are carried in a different direction from the plume axis (see e.g. Taddeucci et al. 2011).

### Implications of modelled plume properties on lightning rates

If ice-based charging is influencing the electrification of the plume then, in addition to water freezing temperatures within the plume, saturation of water vapour is also required in order to obtain the necessary ice and graupel. Figure 7 shows that increased lightning rates are associated with the availability of condensed water and plume top temperatures of around −20°C or lower. In particular, the onset of lightning on 11–12 May occurs as plume top temperature decrease towards −20°C and there is a concomitant abrupt transition from unsaturated plumes to plumes which contain a substantial quantity of condensed water, conditions favourable for a mixed phase of liquid water and ice. Prior to 11 May, when lightning was seldom detected, there are few occasions when both condensed water is found in the plume and plume top temperatures approach the freezing temperature. The few lightning events detected on 6 May do not appear to be associated with condensed water and cold temperatures, but conditions allowing ice formation are predicted to occur in the plume 6 hours before the lightning is detected.

**Fig. 8:**
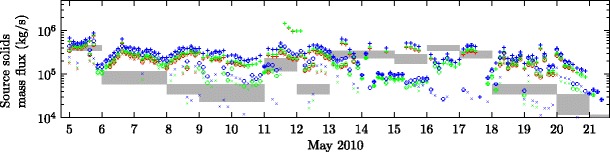
Variation in the source solids mass flux during the second explosive phase of the 2010 eruption of Eyjafjallajökull, as estimated by Gudmundsson et al. (2012) (shaded areas) and predicted by the plume model (points). Model predictions with source conditions determined using the median plume height (∘), maximum plume height (+ ) and minimum plume height (×) are shown, with red points denoting predictions using the ‘hot and dry’ source conditions, green points denoting ‘intermediate’ conditions, and blue points denoting ‘cool and wet’ conditions

As there is no correlation between lightning discharge rates observed by the LMA and the source mass flux, it is not apparent that lightning rates could be used here as a means of determining the source mass flux. However, the plume model provides estimates of the source mass flux (Fig. 8) which compare quite well with estimates obtained from direct sampling of tephra deposits (Gudmundsson et al. 2012), although the estimates are sensitive to the plume height, and the range of plume heights observed during a three-hour interval can lead to order-of-magnitude differences in the source mass flux estimated by the model. The model estimates of the source mass flux are also dependent on the source conditions used, with higher source mass flux required for the ‘cool and wet’ conditions compared to ‘hot and dry’ conditions due to the reduced thermal energy content of the modelled erupted material (Fig. 8).

Variations in the rates at which volcanic lightning discharges occurred at Eyjafjallajökull are seen to be only weakly affected by the plume dynamics and atmospheric conditions. The highest rates of detection of lightning discharges by ATDnet are found during periods of low atmospheric temperature (Arason et al. 2011a), low plume top temperature, and when condensation of water vapour occurs in the plume. However, the LMA detects high discharge rates during both periods when the plume top temperature is relatively warm and periods when no condensation is expected. The sudden onset of sustained lightning activity on the evening of 11 May 2010 cannot be fully explained. While the model predicts the initiation of condensation that is coincident with onset of lightning activity, the plume top temperature does not fall below the temperature at which substantial ice formation would occur. However, the prediction of plume top temperature is sensitive to the plume height and so strongly effected by uncertainties in the radar-derived plume heights, but the radar data currently represents the only continuous record of plume heights available during the eruption.

### Charge structure within the plume

**Fig. 9:**
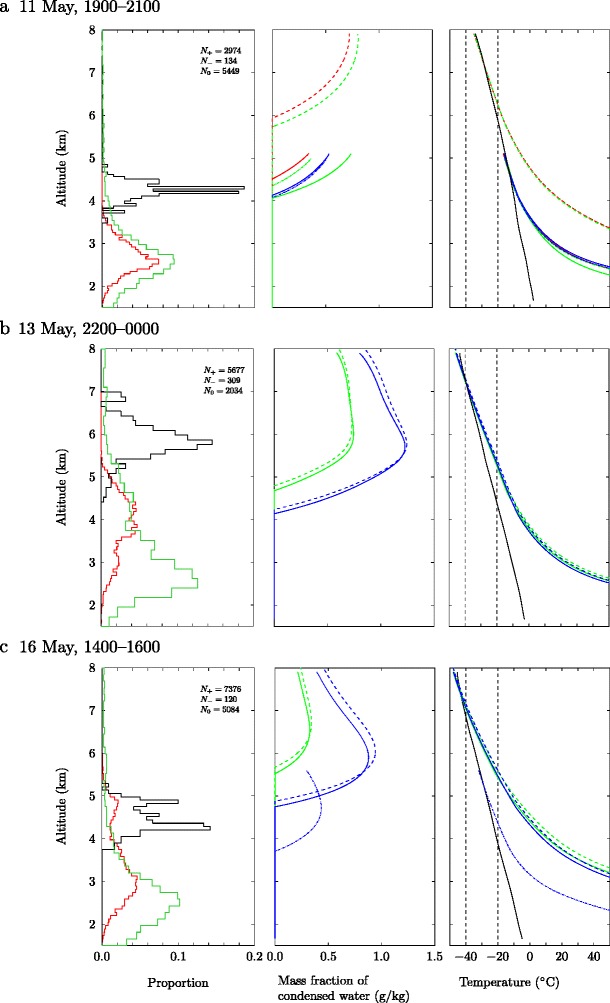
A comparison of the charge structure in the plume (left panels), as inferred from LMA observations, with the water content (centre panels) and temperature (right panels) in the plume, predicted using the integral plume model, for three two-hour intervals where a dipolar structure was observed: (a) 11 May, 1900–2100; (b) 13 May, 2200 – 14 May, 0000; (c) 16 May, 1400–1600. Plume model predictions (centre and right panels) are made at the mid-point time in the interval with interpolated NWP meteorology, using ‘hot and dry’ (red), ‘intermediate’ (green), and ‘cool and wet’ (blue) source conditions, and matching the plume top height predicted by the model to the median (solid lines), maximum (dashed lines) and minimum (dot-dash lines) of the radar observed plume heights within the two-hour interval. (Left panels) Histograms showing the proportion of VHF sources associated with regions of positive charge (red), negative charge (black) or regions where the charge cannot be determined (green) occurring at a specified altitude. The number of VHF sources used in the charge structure analysis is shown, with *N*_+_ denoting sources associated with positive charge, *N*_−_ denoting sources associated with negative charge, and *N*_0_ giving the number of sources for which the charge cannot be determined. (Centre panels) The mass fraction of condensed water as a function of height. (Right panels) The plume temperature as a function of height. The ambient atmospheric temperature as a function of height (black solid lines). Note the plume temperature greatly exceeds 50°C near the vent. The temperatures at which ice formation is expected, −20°C, and at which all condensed water is expected to be ice, −40°C, are marked

**Fig. 10:**
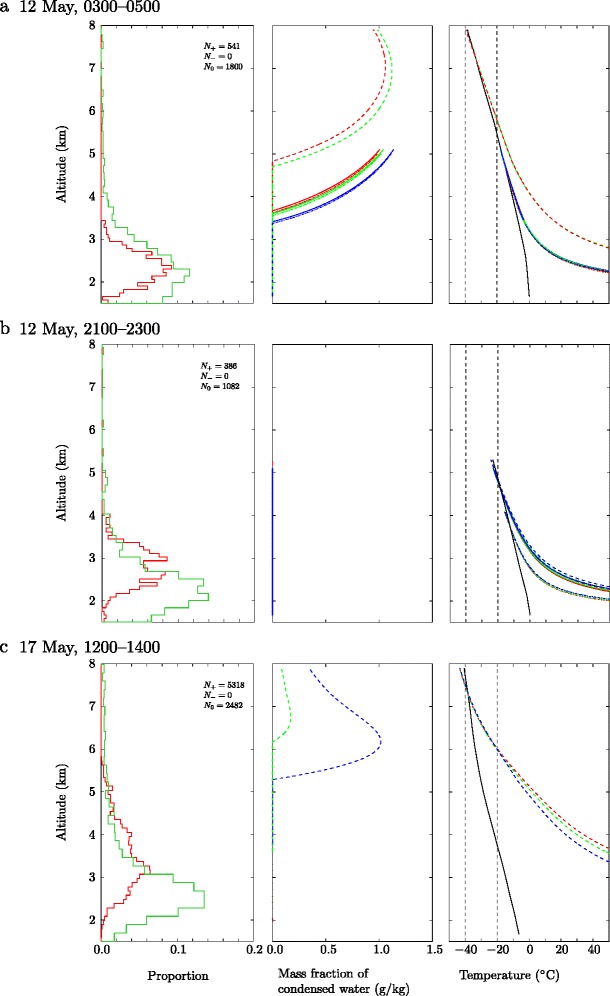
A comparison of the charge structure in the plume (left panels), as inferred from LMA observations, with the water content (centre panels) and temperature (right panels) in the plume, predicted using the integral plume model, for three two-hour intervals where a monopole charge structure was observed: (a) 12 May, 0300–0500; (b) 12 May, 2100 – 2300; (c) 17 May, 1200–1400. See caption in Fig. 9 for details

The LMA data from Eyjafjallajökull 2010 is dominated by discharges near the vent which show a net positive charge carried on volcanic ejecta (Figs. 9 and 10). However, on occasion, a negative-over-positive charge dipole structure is found.

On the night of 13–14 May 2010 there is a prolonged period for which the negative-over-positive charge structure is found (Fig. 2a and Behnke et al. 2014). The negative charge region coincides with the level at which condensation is predicted to occur in the plume when either the median or maximum radar-derived plume height is used and either the ‘cool and wet’ or ‘intermediate’ source conditions are adopted (Fig. 9b). (Note the minimum plume height measured by the radar is at a much lower altitude than the highest VHF source detected, and the model is unable to match the radar heights with a physically plausible source velocity when the ’hot and dry’ conditions are used.) Furthermore, the temperature in the plume above the level of condensation falls below −20°C, and reaches −40°C close to the plume top (Fig. 9b), so substantial ice formation is expected to occur within the plume leading to mixed phase conditions. These conditions are conducive for thunderstorm-style ice-based charge separation. Though an ice-based charging mechanism is not required to produce a dipole charge structure, the model data can be used to assess how ice-based charging may have affected the charge structure.

In general, there are two possibilities. One is that the negative charge is carried on graupel, which implies that there would have been an upper positive charge region carried on ice crystals above approximately 7 km altitude that was not significant enough to show up in the lightning data. Alternatively, the negative charge is carried on ice crystals, which implies that some of the positive charge inferred from the lightning data is carried on graupel. Specifically predicting the polarity acquired by graupel is not possible, however, given that the presence of ash presents a different chemical situation than that of a pure water cloud. Furthermore, the charging of ice and graupel is related to the history of the formation and transport of each species in the moist environment and thus requires a detailed description of the microphysical processes in the turbulent convective flow.

During 1900–2100 on 11 May 2010, the negative-over-positive dipole was observed intermittently (Behnke et al. 2014). The plume model applied during this interval, with either the median or minimum plume height, predicts an increasing condensed water content in the upper part of the plume during this period (Fig. 9a), with the altitude at which condensation occurs falling over time (not shown). However, the model does not predict plume top temperatures below −20°C, although the plume top temperature reaches −17°C during this period so some freezing is possible (Durant et al. 2008). Therefore, a small increase in the plume height above 5km is likely to result in the presence of ice in the plume. Indeed, taking the maximum plume height, the temperature is predicted to fall below −20°C, although the plume height is then significantly higher than the altitude of the highest VHF source (Fig. 9a). Note, the radar record does not contain plume heights for the period 1900–2100 on 11 May, so the nearest available datum (at 1500 on 11 May) is used.

While a negative-over-positive charge structure was observed on 16 May 2010 between 1400 and 1600, the dipole structure only occurred sporadically (Behnke et al. 2014). The LMA dataset records twenty discharges in this interval, of which only two show the dipole charge structure, and there is evidence of changing polarity of the upper region with time (Behnke et al. 2014). This is reflected in the histograms of the altitude at which charge is found in Fig. 9c as the secondary peak in the histogram associated with positively-charged sources that occurs at altitudes where negative charge is also found. The plume model predicts that condensation of water vapour occurs at an altitude coincident with the negative charge region when the minimum radar plume height and ‘cool and wet’ conditions are used (Fig. 9c), but the mass fraction of condensed water in the plume is smaller here than on 13 May 2010 (Fig. 9b). If the median plume height in the radar record is used, more substantial condensation is predicted to occur but at altitudes above the region of net negative charge. Therefore, while the temperature in the plume would allow for freezing of water, variations in the plume height could result in substantial changes in the amount of condensed water in the plume. If the ‘hot and dry’ source conditions are used, the plume heights cannot be matched with physically plausible source velocities.

We consider next two periods on 12 May 2010 for which a positive-charge monopole structure was inferred from the charge structure analysis (Fig. 10a and b). For 0300–0500, there was a high rate of lightning discharges observed by both the LMA and ATDnet. In comparison, for 2100—2300, the LMA detected numerous discharges but ATDnet did not record any lightning events. The model predictions for 0300–0500 (Fig. 10a) show substantial condensation of water, but a plume temperature that remains above −20°C (unless the maximum of the radar plume heights is used, but this height is significantly higher than the altitude of the highest VHF source), and therefore we do not expect ice formation in the plume. For the period 2100–2300 the model does not predict condensation occurring in the plume for any of the source conditions used (Fig. 10b), as the plume remains unsaturated with respect to water vapour. Therefore, although the plume top temperature falls below −20°C, we do not expect ice formation within the plume.

For the period 1200–1400 on 17 May 2010 the charge structure analysis reveals evidence of a positive monopole only (Fig. 10c and Behnke et al. 2014). The rise height of the plume calculated by the model can be matched to only the maximum height in the radar record during this interval and then the model predicts both water condensation and temperatures allowing ice formation but at altitudes above the VHF sources detected by the LMA (Fig. 10c). This could indicate that a negative charge developed from the charge separation expected in ice-based charging in the upper part of the plume but was not sufficient to overcome the positive charge carried on volcanic ejecta and therefore this region was not electrically active.

The comparison of charge structure determined from analysis of LMA discharges with model-derived predictions of the profiles of temperature and condensed water content within the plume suggest the development of the negatively charged region at high altitudes is closely linked to the formation of ice near the plume top. However, if ice formation and ice-based charging is indeed responsible for the development of the dipolar structures then it is having only a marginal effect on the overall electrification of the plume, which was dominated by silicate-based charging mechanisms (Aizawa et al. 2010; Cimarelli et al. 2014; Behnke et al. 2014).

## Concluding remarks

The three-dimensional spatial location of volcanic lighting that can be obtained from an LMA at a volcano can provide a snap-shot of the trajectory and growth of volcanic plumes during eruptions (Thomas et al. 2007; Thomas et al. 2010; Behnke et al. 2013; Behnke et al. 2014). In addition to the direct plume monitoring value of these observations, comparisons with predictions from models describing plume dynamics can provide additional estimates of the volcanic source conditions. Furthermore, unlike single-value observations, the spatially extensive LMA observations provide a dataset that allows the predictions of modelled plume trajectories to be assessed. We have demonstrated that an integral plume model that incorporates meteorological profiles (albeit at a single location) can provide reasonable predictions of the plume trajectory and growth. Our comparison of the altitude at which VHF sources occur to our model predictions suggest that charge becomes localized on the lower plume edge for the weak volcanic plume studied, possibly due to particle fallout. While our comparison here is only qualitative, quantitative comparisons with the data are possible.

We have reanalyzed the observations of volcanic lightning during the second explosive phase of the 2010 eruption of Eyjafjallajökull. Substantially fewer lightning discharges are detected by the long-range ATDnet system in comparison to the proximally deployed LMA. We have shown that the number of discharges detected by ATDnet are influenced by the atmospheric conditions, in particular the condensation of water vapour at temperatures where mixed water and ice phases are expected. The number of lightning discharges observed by the LMA are less affected by atmospheric conditions. The sporadic appearance of the freezing conditions in the Eyjafjallajökull plume suggest that plume monitoring based on lightning detection systems that predominately detect high-current (or extensive) discharges, such as ATDnet, could fail to locate volcanic plumes during periods of unfavourable meteorological and/or volcanologicalconditions.

The analysis of LMA discharges reveals the occasional appearance of negative-over-positive charge dipole structures. Our model results show the altitude of the negatively charged region is closely connected to the altitude at which condensation of water vapour is predicted to first occur. This suggests the formation of ice is influencing the development and transport of electrical charge within the plume.

## Figures and Tables

**Fig. 11:**
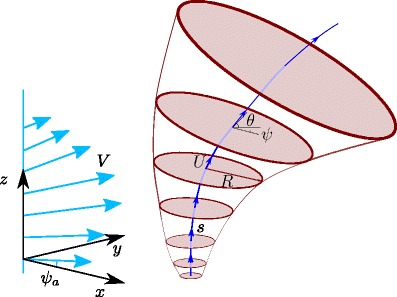
A model of a volcanic plume in a cross-wind. A Cartesian coordinate system is fixed with *x* denoting the East-West (longitudinal) coordinate, *y* denoting the North-South (latitudinal) coordinate and *z* denoting the vertical coordinate (altitude). Equations describing the plume dynamics are derived in a plume-centered coordinate system, with *s* denoting the curvilinear distance (arclength) from the vent along the plume axis, *𝜃*(*s*) denoting the angle of the centerline with respect to the horizontal, and *ψ* the angle of the trajectory in the *xy*-plane. A cross-section of the plume normal to the centerline is circular with radius *L*(*s*). The centerline speed of the plume is denoted by *U*(*s*). The wind speed is denoted by *V*(*z*), with the angle *ψ*_*a*_ denoting the angle to which the wind blows

## Appendix

We present the governing equations used in the integral model. Full details of the model derivation and assumptions are given in Woodhouse et al. (2013).

The coordinate system employed is shown in Fig. 11. The variables and parameters appearing in the model are defined in Tables 3 and 4, respectively.

**Table 3:** Variables in the plume model

Variable	Symbol	Comment
Distance from vent along plume centreline	*s*	
Longitudinal coordinate	*x*	
Latitudinal coordinate	*y*	
Vertical coordinate (above sea level)	*z*	
Mass flux	*Q*	*Q* = *ρUR* ^2^
Source mass flux	*Q*_0_	
Axial momentum flux	*M*	*M* = *ρU* ^2^ *R* ^2^
Plume radius	*R*	
Centreline speed	*U*	
Entrainment velocity	*U*_*e*_	
Plume temperature	*T*	
Plume density	*ρ*	
Mass fraction of gas	*n*	Mass fraction of solids = 1 − *n*
Mass fraction of gas at vent	*n*_0_	
Mass fraction of total water (vapour	*ϕ*	
and condensed phases)		
Mass fraction of water vapour	*ϕ*_*v*_	
Mass fraction of condensed water	*ϕ*_*w*_	*ϕ*_*w*_ = *ϕ* − *ϕ*_*v*_
Mass fraction of gas that is water vapour	*w*	*w* = *ϕ*_*v*_/*n*
Heat capacity of plume	*C*_*p*_	
Gas constant of plume	*R*_*g*_	
Angle of centreline to horizontal	*𝜃*	
Angle of centreline to *x*-axis	*ψ*	
Angle from *x*-axis to which wind blows	*ψ*_*a*_	
Wind speed	*V*	
Pressure	*P*	Plume pressure equals atmospheric
		pressure
Saturation vapour pressure	*e*_*s*_	
Atmospheric temperature	*T*_*a*_	
Gas constant of atmosphere	*R*_*ga*_	
Heat capacity of atmosphere	*C*_*A*_	
Atmospheric air density	*ρ*_*a*_	
Atmospheric relative humidity	*R*_*H*_	
Atmospheric specific humidity	*ϕ*_*a*_

**Table 4:** Parameters in the plume model

Parameter	Symbol	Value
Density of liquid water	*ρ*_*w*_	1000 kg/*m* ^3^
Density of solid pyroclasts	*ρ*_*s*_	1200 kg/*m* ^3^
Entrainment coefficient in absence of wind	*k*_*s*_	0.09
Entrainment coefficient due to wind	*k*_*w*_	0.9
Gas constant of dry air	*R*_*a*_	285 J/K/kg
Gas constant of water vapour	*R*_*v*_	462 J/K/kg
Gravitational acceleration	*g*	9.81 m/*s* ^2^
Latent heat of vaporization at 273 K	*L*_*c*0_	2.5×10^6^ J/kg
Specific heat capacity of dry air	*C*_*a*_	998 J/K/kg
Specific heat capacity of liquid water	*C*_*w*_	4200 J/K/kg
Specific heat capacity of solid pyroclasts	*C*_*s*_	1617 J/K/kg
Specific heat capacity of water vapour	*C*_*v*_	1850 J/K/kg

Mass conservation:

$$\frac{\mathrm{d} Q}{\mathrm{d} s} = 2\rho_{a} U_{e}\frac{Q}{\sqrt{\rho M}}.$$

*z* component of momentum conservation:

$$\frac{\mathrm{d}}{\mathrm{d} s}\left(M\sin\theta\right) = \left(\rho_{a}-\rho\right)g\frac{Q^{2}}{\rho M}.$$

*x* component of momentum conservation:

$$\frac{\mathrm{d}}{\mathrm{d} s}\left(M\cos\theta\cos\psi\right) = 2\rho_{a}\frac{Q}{\sqrt{\rho M}}U_{e} V\cos\psi_{a}.$$

*y* component of momentum conservation:

$$\frac{\mathrm{d}}{\mathrm{d} s}\left(M\cos\theta\sin\psi\right) = 2\rho_{a}\frac{Q}{\sqrt{\rho M}}U_{e} V\sin\psi_{a}.$$

Energy conservation:

$$\begin{array}{*{20}l} & \frac{\mathrm{d}}{\mathrm{d} s}\left(Q\left(C_{p}T + \frac{U^{2}}{2} + gz\right)\right) \\ & \quad = 2\rho_{a} U_{e} \frac{Q}{\sqrt{\rho M}}\left(C_{A}T_{a} + \frac{{U_{e}^{2}}}{2} + gz\right) \\ & \quad + L_{c0}\frac{\mathrm{d}}{\mathrm{d} s}\left(Q\left(\phi-\phi_{v}\right)\right). \end{array}$$

Conservation of total water:

$$\frac{\mathrm{d}}{\mathrm{d} s}\left(Q\phi\right) = 2\rho_{a} U_{e} \frac{Q}{\sqrt{\rho M}}\phi_{a}.$$

Plume bulk density:

$$\frac{1}{\rho} = \frac{nR_{g}T}{P} + \frac{\phi_{w}}{\rho_{w}} + \frac{1-n-\phi_{w}}{\rho_{s}}.$$

Plume heat capacity:

$$\begin{array}{*{20}l} &C_{p} = n\left(wC_{v} + \left(1-w\right)C_{a}\right) + \phi_{w}C_{w} \\ &\quad + \left(1-n-\phi_{w}\right)C_{s}. \end{array}$$

Plume gas constant:

$$R_{g} = wR_{v} + \left(1-w\right)R_{a}.$$

Mass fraction of gas:

$$n = 1 - \phi_{w} - \left(1-n_{0}\right)\frac{Q_{0}}{Q}.$$

Mass fraction of liquid water:

$$\phi_{w} = \phi - \phi_{v}.$$

Mass fraction of water vapour:

$$\phi_{v} = nw.$$

Mass fraction of gas that is water vapour when unsaturated:

$$w = \frac{\phi}{n}.$$

Mass fraction of gas that is water vapour when saturated:

$$\frac{wR_{v}}{wR_{v}+\left(1-w\right)R_{a}}P = e_{s}\left(T\right).$$

Atmospheric density (equation of state):

$$\rho_{a} = \frac{P}{R_{ga}T_{a}}.$$

Atmospheric gas constant:

$$R_{ga} = \phi_{a}R_{v} + \left(1-\phi_{a}\right)R_{a}.$$

Heat capacity of atmosphere:

$$C_{A} = \phi_{a}C_{v} + \left(1-\phi_{a}\right)C_{a}.$$

Specific humidity of atmosphere:

$$\phi_{a} = \frac{R_{H}e_{s}\left(T_{a}\right)R_{a}}{R_{v}P - R_{H}e_{s}\left(T_{a}\right)\left(R_{v}-R_{a}\right)}.$$

Saturation vapour pressure (from Alduchov and Eskridge 1996):

$$e_{s}(T) = 610.94\exp\left(\frac{17.625\left(T - 273.15\right)}{T-30.01}\right).$$

*x* coordinate of plume centreline:

$$\frac{d x}{d s} = \cos\theta\cos\psi.$$

*y* coordinate of plume centreline:

$$\frac{d y}{d s} = \cos\theta\sin\psi.$$

*z* coordinate of plume centreline:

$$\frac{d z}{d s} = \sin\theta.$$

The entrainment formulation adopted by Woodhouse et al. (2013) is that of Hewett et al. (1971),

$$U_{e} = k_{s}\left|U - V\cos\theta\right| + k_{w}\left|V\sin\theta\right|,$$

where *k*_*s*_ and *k*_*w*_ are dimensionless entrainment coefficients.

The plume model adopts top-hat profiles to describe the variation of quantities within the plume (Morton et al. 1956), while experiments show Gaussian profiles more closely resemble the observed variations (Kaye 2008). In comparisons of the plume width predicted by the model to LMA observations (Figs. 5 and 6) an equivalent Gaussian plume width, *R*_*G*_, is used, with $R_{G}=R/\sqrt {2}$ (Kaye 2008).
